# The Loppered Milk Diet*Read before the Medical Association of Central New York, Oct. 18, 1917, at Auburn.

**Published:** 1917-12

**Authors:** Geo. N. Jack

**Affiliations:** Buffalo, N. Y.


### The Loppered Milk Diet.*

### Including References to its Benefits in Post-Operative Disturbances and Metabolic Defectives.

DR. GEO. N. JACK, Buffalo.

The value of a loppered or thick milk diet in prolonging life, restoring health and preventing sickness has long been known. Sour milk has been one of the principal features of the diet in Turkey, Roumania, Bulgaria and Servia for many centuries. In about 1902, Prof. Massol of Geneva, noticed that the peasants of these countries lived to an astonishing age. A count of them showed that over five thousand of the peasants of Bulgaria at one time were over one hundred years old. They lived on Yoghourt, a preparation of sour milk. It was their principal article of food and each ate over a quart of it a day.

Subsequent experiments showed Prof. Massol that what made sour milk so healthful was the presence of lactic acid. The lactic acid results from the fermentation. This acid owing to its antiseptic action, tends to prevent diseases originating in the gastro-intestinal tract.

If we divide the first essentials for the health and life of all animals into quarters, we would have (a) inherited blood vitality, and metabolism, (b) air, (c) food and water, (d) suitable environment, including weather and climatic conditions. It would follow then that all functional sickness is caused by a perversion of one or more of the above mentioned first essentials of health. This would give to diet one-fourth of the responsibility in preventing and curing functional sickness.

The blood must draw by metabolism from our food and air, under a suitable environment, what is necessary for life and health.

Milk contains all the minerals, chemicals, substances or elements necessary for the maintenance of perfect health. If an individual then does not thrive on a milk diet, the milk is either poisoned by the contents of the gastro-intestinal tract or the blood is too feeble to metabolize it.

Owing to the fact that loppered milk has fermented or soured, it is practically digested before it is eaten, therefore it constitutes an ideal predigested food. Loppered milk, being a predigested food, is metabolized or transformed into life energy or vital force and muscle with but little tax on the

*Read before the Medical Association of Central New York, Oct. 18, 1917, at Auburn.

digestive organs. This renders loppered milk an ideal diet in cases of feeble digestion or metabolism.

Auto-intoxication or self poisoning from the gastro-intestinal tract is one of the chief causes of functional sickness. The loppered milk diet, therefore, through its antiseptic action, tends to prevent sickness as well as to cure it.

While on a strict loppered milk diet, the stools as a rule are practically odorless, neutral in reaction and show no, or but little evidence of fermentation or putrefaction.

My five years experience with the loppered milk diet has demonstrated to me its efficiency in the prevention of seasonal, functional, food and weather sicknesses such as summer autumnal coryza, asthma, digestive or diarrhoeal troubles and rheumatism; also the winter spring functional food weather sicknesses as colds, lagrippe, bronchitis, pneumonia, eczema, sugar diabetes and asthma.

My method of obtaining loppered milk has been as follows: The first essential is to obtain rich milk from healthy cows that have been properly fed and cared for. After milking the milk should be carefully strained and cooled with a milk cooler. After cooling the milk should be bottled in a clean sanitary room and stored in a coagulating room such as a light clean cellar with a temperature of about 60 degrees. This cellar should be well ventilated and used for no other purpose.

The milk after being bottled should stand without caps on, thus allowing the milk to lopper in the open air. After the milk has coagulated, the bottles should be capped and delivered to the consumer's house. The consumer should then remove the caps and store the bottles in an ordinary pantry where the loppered milk will keep from a week to ten days.

Those on the loppered milk diet should keep at least ten bottles ahead, for two reasons, first the older the better and second so that they will not have to skip a feeding as it is a great disappointment for one on this diet to be compelled to go without it.

Loppered milk, when properly loppered, separates into whey at the bottom, curds in the middle and cream on the top. From four to six ounces of whey generally forms in a quart of good rich milk. A colony of mould generally forms on top of the cream which should be removed before emptying the bottle.

Loppered milk seems to have a better flavor if loppered in paraffined spruce wood fibre containers in place of the glass bottles.

My method of prescribing the loppered milk diet is as follows: I have my patients procure a quart butter churn in which a quart of the loppered milk can be churned up at one time. Two or three minutes of brisk churning of this loppered milk gives it an even, rich, creamy consistency, with a delicious flavor that agrees with the most delicate stomachs.

Patients that are greatly emaciated or badly reduced in health, I generally restrict to a well cooked fruit, cereal diet in conjunction with the loppered milk. I endeavor to have them take about a pint of freshly churned loppered milk through a tube with buttered toast or a cheese or butter sandwich with a baked apple every two hours.

All of my emaciated cases taking the loppered milk have done so in conjunction with indicated medicinal treatment. After one of two weeks treatment with the restricted loppered milk diet, my emaciated patients that are doing well are generally put on a more liberal diet, having three regular meals a day. At this period I try to have my patients take a pint of loppered milk at meals and between meals, or about five pints a day. Many of my patients have averaged six pints of loppered milk a day for years.

A knowledge of what has now come to be known as anaphylaxis has added a new significance to the diet question.

In my Pathology of Asthma that I wrote in 1901 or one year before Richet coined the term anaphylaxis, I gave the following definition of asthma:

It will be seen, then, that asthma is not a disease by itself having a well-established entity, but that it is only a symptom, a part of a vicious circle, or an abnormal biochemical and complex pathological process, originating usually in the intestinal canal, through a longstanding intestinal indigestion and toxemia, with faulty absorption and metabolism, producing a toxic or lymphagenous chyle that generates an unstable blood, characterized by its extremely varied, numerous and alarming paroxysmal, morphologic changes, often alternating between a lymphocytosis, an intestinal toxemic leucocytosis, or a marked anemia; accompanied anatomically by a hyperplasia of the lymphatic and glandular structures and clinically by a most wretched and agonizing dyspnea, This definition makes it plain that the one characteristic pathological feature of asthma is the unstableness of its blood.

This definition shows the relationship between asthma and anaphylaxis and also shows that asthma is not due to anaphylaxis but to an unstable blood. The unstable blood of the asthmatic is hypersusceptible and sensitized to numerous food and weather conditions which causes it readily to undergo the anaphylactic phenomenon.

The blood of the asthmatic between attacks generally appears quite normal, but just before or during an attack we can characteristically detect trouble. The toxic leucocytic blood, for instance, just before or during an attack, as a rule flows in small, slow forming black drops, with a high viscosity, a rapid coagulation and a thick consistency. The black drop with its diminished vitality slowly enters the test paper and shows a high hemoglobin scale. If a few drops of hydrogen peroxide be added to a fresh drop of this toxic blood on a cover glass, it generally gives a rapid dirty brown reaction while that of normal blood is pink. The leucocytes are increased and show an amyloid degeneration. The oesinophile cells are increased. Often 20 to 50% of the erythrocytes are star shaped or irregular, showing a granular disintegration. The roloux formation is generally absent. It as a rule requires an extraordinary amount of pressure on the cover glass to differentiate the cells of this toxic blood, as they tend to form in an opaque, shapeless adherent mass.

During the last twenty years in conjunction with a general practice I have made a study of 924 cases of asthma and their relation to food and weather conditions and I have come to the following conclusions:

I. The cause of asthma and summer autumnal coryza is an unstable blood.

II. Owing to the unstability of the blood of the asthmatic and summer autumnal coryza subjects, it is rendered hypersusceptible and sensitized to numerous metabolic, gas, weather and food conditions.

III. I consider the blood as the biochemical medium between the weather on the lung skin and the food on the gastro-intestinal side.

IV. The hypersusceptible and sensitized blood of the asthmatic and the summer autumnal coryza subject is sufficiently disintegrated to produce attacks (1) automatically, (2) by unfavorable food conditions, (3) by unfavorable weather conditions, (4) by toxic gas conditions, and (5) by a combination of all of the above conditions. These have occurred in about the following ratio:

(a) In about one fifth of all my asthmatics their unstable blood has been automatically, biochemically, metabolically, glandularly or functionally periodically disintegrated sufficiently to produce an attack of asthma.

(b) In one fifth of all asthmatics their unstable blood is sufficiently disintegrated by unfavorable food conditions to produce attacks of asthma.

(c) In about one fifth of all my asthmatics and all summer autumnal coryza subjects their unstable blood has been sufficiently disintegrated by unfavorable weather conditions to produce attacks of coryza and asthma.

(d) In one fifth of all asthmatics their unstable blood is sufficiently disintegrated by toxic gas conditions to produce attacks of asthma, as swamp gas, animal excreta gas, gas from decomposing vegetation, night ground gas, burning sulphur gas, etc.

(e) In one fifth of all asthmatics their unstable blood is sufficiently disintegrated to produce attacks of asthma by a combination of two or more of the above mentioned automatic, food, weather and gas blood disintegrating agents.

V. The unstable blood of the asthmatic runs low in certain minerals or chemicals that must be supplied in the form of medicines until it can draw the same from a prescribed diet that is rich in the minus minerals or chemicals.

VI. The treatment of asthma could be likened to the unlocking of a combination lock; one may know all the combinations but one and yet he will not be able to unlock the lock until he determines the missing combination.

(To be concluded in January issue.)

---

**Experimental Typhus.** M. H. Neill, Passed Asst. Surgeon, U. S. P. H. S., Pub. Health Reports, July 13, alludes to the striking similarity between the lesions of typhus and Rocky Mountain spotted fever, though their non-identity is apparently demonstrated by amynologic tests. 26 of 37 Guinea pigs, killed at the height of febrile reaction after intraperitoneal injection with Mexican typhus virus showed lesions, consisting of petechiae or considerable haemorrhages in the cremasteric fascia and immediately beneath the visceral laminae of the tunica vaginalis, the skin of the scrotum appearing normal. These are milder in character than similar lesions of Rocky Mountain fever.

---

**Multiple Intracranial Propectiles.** V. Morax, Ann d'Oculistique, May, describes a military case. Mental hebetude and diminution of vision were noted almost immediately but improvement occurred and no ocular lesions were found at the first examination. By X-rays and at autopsy, three fragments were demonstrated, one in the right occipital lobe, with thrombosis, another which had lacerated the same lobe and had been arrested by the tentorium cerebelli, a third had reached the same lobe, giving rise to a purulent leptomeningitis which resulted in death. Oscular lesions observed during the course of the process were: homonomous hemianopsia, neuro-paralytic keratitis, paralysis of the dextro-gyric muscles.

